# Proposal of a new conceptual gait model for patients with Parkinson’s disease based on factor analysis

**DOI:** 10.1186/s12938-019-0689-3

**Published:** 2019-06-03

**Authors:** Ilaria Arcolin, Stefano Corna, Marica Giardini, Andrea Giordano, Antonio Nardone, Marco Godi

**Affiliations:** 1 Istituti Clinici Scientifici Maugeri Spa SB (IRCCS), Pavia, Italy; 2 Department of Clinical-Surgical, Diagnostic and Pediatric Sciences, University of Pavia, Pavia, Italy

**Keywords:** Gait, Parkinson’s disease, Model, Factor analysis

## Abstract

**Background:**

Gait impairment is a risk factor for falls in patients with Parkinson’s disease (PD). Gait can be conveniently assessed by electronic walkways, but there is need to select which spatiotemporal gait variables are useful for assessing gait in PD. Existing models for gait variables developed in healthy subjects and patients with PD show some methodological shortcomings in their validation through exploratory factor analysis (EFA), and were never confirmed by confirmatory factor analysis (CFA). The aims of this study were (1) to create a new model of gait for PD through EFA, (2) to analyze the factorial structure of our new model and compare it with existing models through CFA.

**Results:**

From the 37 variables initially considered in 250 patients with PD, 10 did not show good-to-excellent reliability and were eliminated, while further 19 were eliminated after correlation matrix and Kaiser–Meyer–Olkin measure. The remaining eight variables underwent EFA and three factors emerged: pace/rhythm, variability, and asymmetry. Structural validity of our new model was then examined with CFA, using the structural equation modeling. After some modifications, suggested by the Modification Indices, we obtained a final model that showed an excellent fit. In contrast, when the structure of previous models of gait was analyzed, no model achieved convergence with our sample of patients.

**Conclusions:**

Our model for spatiotemporal gait variables of patients with PD is the first to be developed through an accurate EFA and confirmed by CFA. It contains eight gait variables divided into three factors and shows an excellent fit. Reasons for the non-convergence of other models could be their inclusion of highly inter-correlated or low-reliability variables or could be possibly due to the fact that they did not use more recent methods for determining the number of factors to extract.

## Introduction

Falls are both common and debilitating in patients with Parkinson’s disease (PD). Gait impairment is among the factors contributing to increased risk of falls in PD [1, 2], together with leg muscle weakness and poor balance [2–4]. Indeed, patients with PD are more often admitted to hospital than healthy individuals because of a fall-related injury [5].

Electronic walkways are an easy instrument for assessing gait that provide a large number of spatiotemporal variables of gait. Even if these systems are expensive and led to evaluate gait only in the laboratory context, they have been often used to assess gait of patients with PD in clinical practice [6–9]. In particular, the GAITRite^®^ system was demonstrated to be a reliable instrument [10] and showed an excellent concurrent validity for measuring individual footstep data [11]. However, to date there are no agreed standardised protocols for measuring gait or for selecting which gait variables among the plethora of collected ones are best suited to assess gait in patients with PD. Numerous models of gait were previously developed with the aid of factor analysis to reduce the number of variables collected and to collect them into factors [12–18]. Most of these models were developed in older adults and include from 3 to 5 different factors [12, 13, 16–18]. For instance, Verghese et al. [16–18] identified 3 factors that characterize gait performance in older adults: “rhythm”, “pace”, and “variability”. The “pace” factor, in particular, predicted decline in executive function in older adults [16]. More recently, Hollman et al. [12] divided the spatiotemporal gait variables into the following 5 factors: “rhythm”, “phases of the gait cycle”, “variability”, “pace” and “base of support”. Lord et al. [13] developed a model with a smaller number of variables, and identified similar factors to Hollman’s model that accounted for 79.5% of total variance in test scores: “pace”, “rhythm”, “variability”, “asymmetry”, and “postural control”. Finally, Thingstad et al. [15], after a first selection of gait variables, created a model for older patients with hip fracture based on four factors: “variability”, “asymmetry” and “postural control” and another single factor combining pace and rhythm (“pace/rhythm”).

As regards patients with PD, only Lord et al. [14] created a specific model to evaluate gait in PD with an electronic walkway. Their model is similar to that of healthy subjects except for a few variability parameters (step time, stance time and step width variability) that were allocated in different factors. Other authors [19, 20] created gait models specific for patients with PD, but using inertial sensors rather than an electronic walkway. In particular, Horak et al. [19] used body-worn sensors to determine functional mobility domains for evaluating gait, postural sway, step initiation, turning, and trunk and arm motion when patients were performing an Instrumented Stand and Walk Test. On the other hand, Morris et al. [20] used body-worn sensors for analysing only gait parameter in a controlled and in a free-living environment, finding four factors.

The seven existing models proposed for assessing gait with the electronic walkway were developed in large samples in the case of elderly subjects [12, 13, 15–18], but in the single case of patients with PD the sample size was small (*n* = 121) [14]. In addition, some methodological limitations affected all models: (a) shortcomings in the procedure of exploratory factor analysis (EFA); (b) lack of confirmation of the models through confirmatory factor analysis (CFA).

Regarding the procedure of EFA, in some models [12, 14–18] the reliability of the included variables was low [21, 22] or not yet tested. In addition, in all seven models, only one method of factor determination (Kaiser criterion), when specified, was used despite the recommendation in the literature to apply more than one method [23, 24]. Moreover, none of the studies took into consideration other methods such as parallel analysis and minimum average partial (MAP) rule, considered in the literature to be two of the most accurate approaches for determining the number of factors [25–27].

It appears that all the existing models were defined only through EFA, that serves to determine if numerous measures can be explained by a smaller number of factors, but it cannot verify a model [28, 29]. On the contrary, the models should be confirmed through CFA that is necessary to verify the factor structure of a set of observed variables and test the hypothesis that a relationship exists between observed variables and their underlying latent construct [30].

Given these shortcomings with the existing models, the principal aims of our study were: (a) to create a new model of gait evaluation specific for patients with PD through EFA; (b) to analyze the factorial structure of our new model and compare it with existing models through CFA, to identify the best model suitable for evaluating gait in patients with PD.

## Results

### Inter-trial reliability

**Fig. 1:**
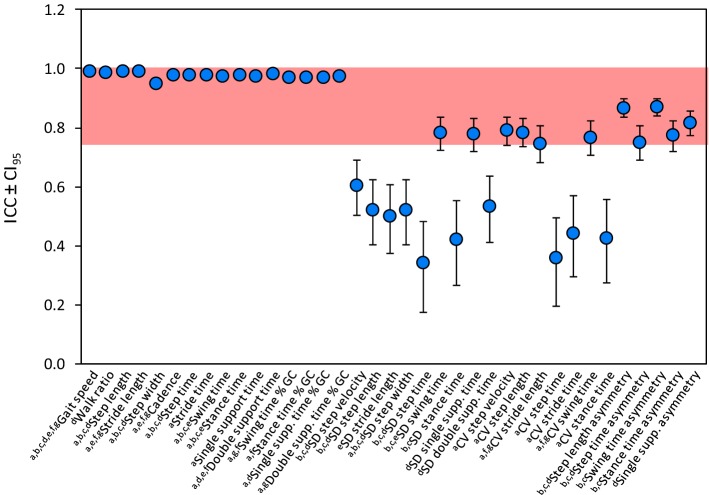
Inter-trial reliability of all gait variables. a. Hollman et al. [12]; b. Lord et al. [13]; c. Lord et al. [14]; d. Thingstad et al. [15]; e. Verghese et al. [16]; f. Verghese et al. [17]; g. Verghese et al. [18]

**Fig. 2:**
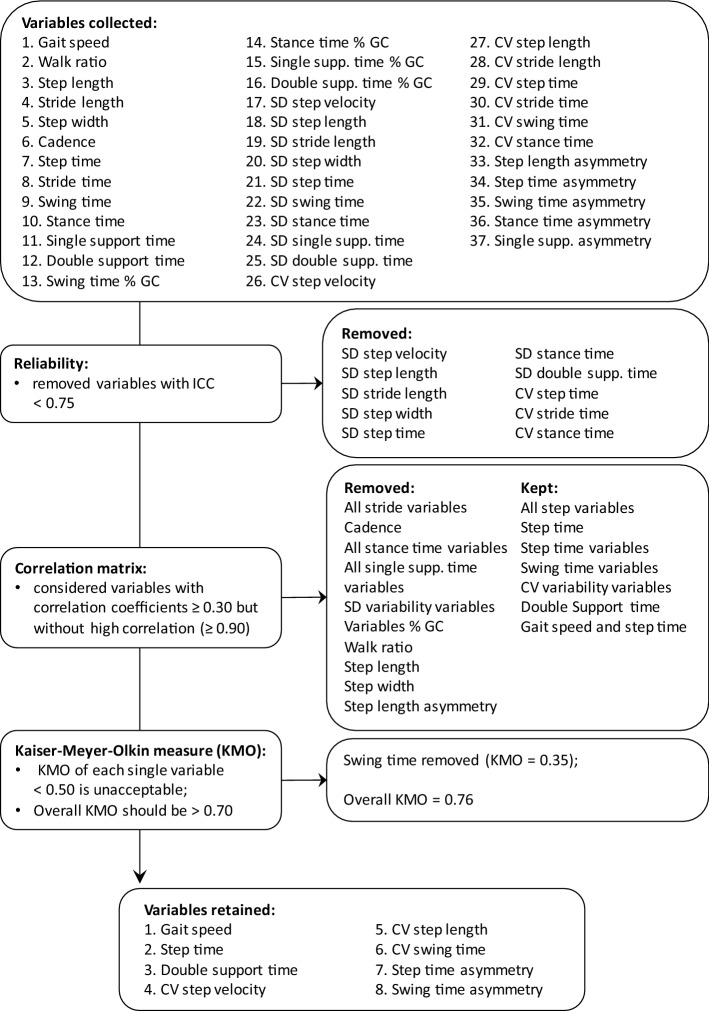
Flow chart describing the initial selection of gait variables

Intraclass correlation coefficients (ICC) values for all 37 gait variables initially considered are shown in Fig. 1. Reliability was excellent for all spatiotemporal gait variables and for all asymmetry variables. On the contrary, the following variability variables showed good-to-fair or poor reliability: SD step velocity, SD step length, SD stride length, SD step width, SD step time, SD stance time, SD double support time, CV step time, CV stride time, CV stance time. These ten variables were excluded from the subsequent analysis (see Fig. 2).

### Correlation matrix

Based on the correlation matrix, the following variables were eliminated from the remaining group of 27 variables: all stride variables, all stance time and single support time variables, all variables expressed as % of gait cycle, walk ratio, step length, step width and step length asymmetry. Since many variability variables expressed as CV showed an adequate reliability, we considered only these ones; therefore, the remaining two variables expressed as SD (swing time and single support time) were eliminated. Walk ratio was eliminated since it was derived from other variables (step length and cadence) [29]. Based on Kaiser-Meyer-Olkin measure (KMO), swing time was eliminated since its value (0.35) did not meet the criteria for analysis set in the Methods. After this selection process, eight variables remained for the factor analysis, with an overall KMO of 0.76, thus enough to produce distinct factors with EFA (see below and Fig. 2).

### Exploratory factor analysis

**Fig. 3:**
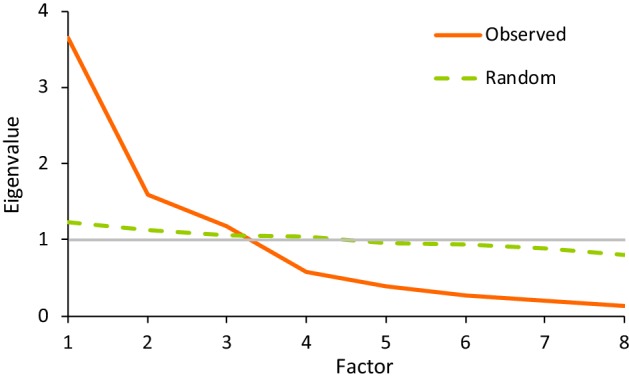
Results of Horn’s parallel analysis on a scree plot. The real data (“observed”) and the random data are presented. Grey line identifies eigenvalue = 1

Principal component analysis (PCA) revealed three factors with an eigenvalue > 1 (Kaiser criterion) (Table 1). These three factors accounted for 80.2% of the total variance in gait performance. In particular, the first factor accounted for 45.6%, the second for 19.9% and the third for 14.7%. The scree plot test was hard to interpret, since it was impossible to determine the point of inflexion (see Fig. 3, “observed” data). Results of Horn’s parallel analysis are shown in Fig. 3. The point at which the line of observed values intersects the line of random uncorrelated data indicates that three factors should be retained, according to the criterion of parallel analysis. Finally, the MAP procedure confirmed that three factors should be extracted.

**Table 1:** Results of principal component analysis

Factor	Eigenvalue	Proportion of variance	Cumulative variance
1	3.645	0.456	0.456
2	1.598	0.199	0.656
3	1.172	0.147	0.802
4	0.577	0.072	0.874
5	0.398	0.049	0.924
6	0.265	0.033	0.957
7	0.206	0.026	0.983
8	0.138	0.017	1

Promax rotation of EFA was conducted on the eight variables considered, according to the three main factors found in the previous PCA analysis. Factors were labelled based on the variables grouped in each factor and in line with previous published models: pace/rhythm, variability and asymmetry (Table 2).

**Table 2:** Factor loading of gait parameters on three factors rotated and extracted by exploratory factor analysis

Variable	1st factor	2nd factor	3rd factor
Gait speed	− *0.674*	− *0.334*	0.044
Step time	*0.918*	− 0.170	0.033
Double support time	*0.908*	0.065	− 0.051
CV step velocity	0.058	*0.738*	− 0.058
CV step length	− 0.061	*0.867*	− 0.008
CV swing time	0.107	*0.603*	0.007
Step time asymmetry	− 0.111	0.066	*0.685*
Swing time asymmetry	0.136	− 0.069	*0.668*

Significant item loading is reported in italic

### Confirmatory factor analysis

**Fig. 4:**
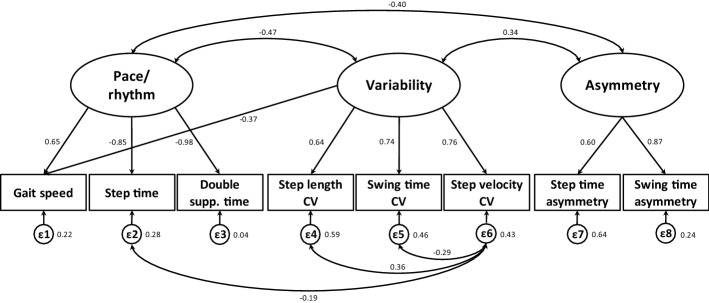
Standardized solution of confirmatory factor analysis for our model. Circles from *ε*1 to *ε*8 represent the measurement errors. One-headed arrows represent correlations while two-headed arrows represent covariance. For each variable, values at the bottom represent errors, while values at the top represent Standardized Regression Weights

According to the results of EFA, our new model was built with structural equation modeling (SEM): it showed an overall mediocre fit to the observed data: *χ*^2^ = 46.44, d*f* = 16, *p* < 0.0005, RMSEA = 0.09 (CI_90_ = 0.06–0.12), CFI = 0.96, TLI = 0.94, SRMR = 0.05. Based on the Modification Indices (MIs), we then tried to improve the fit of the model including the following correlations: step velocity CV correlated with step time, step length CV and swing time CV (Fig. 4). The resulting model (*χ*^2^ = 17.19, d*f* = 13, *p* = 0.16, RMSEA = 0.04 (CI_90_ = 0.00–0.08), CFI = 0.99, TLI of 0.98 and SRMR = 0.03) showed an excellent fit in each index.

**Fig. 5:**
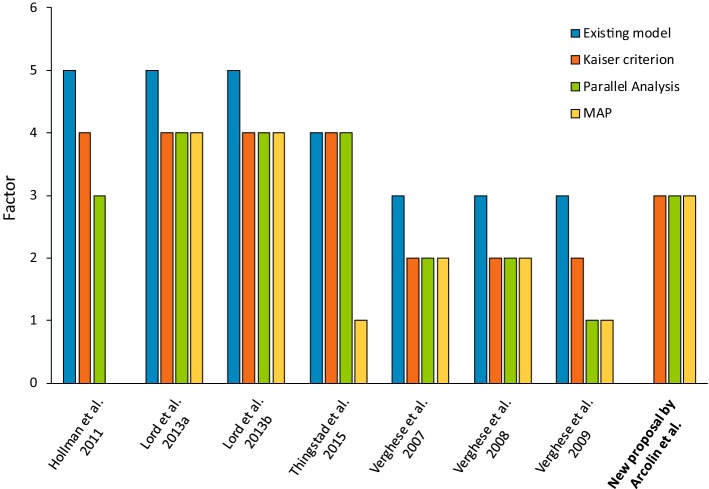
Number of factors in the seven existing models and in our new proposed model. Number of factors are shown as reported in existing models and as calculated in our sample of patients with PD through Kaiser criterion, parallel analysis and MAP

We analyzed with SEM the structure of the other existing gait models [12–18]. No model reached convergence with our sample of patients. Hence, we determined the number of factors through the same methods used for our model (parallel analysis and MAP, in addition to Kaiser criterion). As shown in Fig. 5, the number of factors reported in the existing models was generally different from that found in our analysis. In three of the existing models considered, parallel analysis and/or MAP suggested a smaller number of factors with respect to the Kaiser criterion.

## Discussion

The aims of this study were to create a new model of gait specific for patients with PD and to compare it with existing models in the literature, to identify which is the best model for evaluating gait of patients with PD. To this end, we considered 37 gait variables previously included in models of gait developed in elderly subjects [12, 13, 15–18] and in patients with PD [14]. The structure of our new model was analyzed through CFA. The goodness of fit analysis revealed that the overall content structure of our model (with eight gait variables distributed into three factors) is clear and indicates a good factorial validity. The comparison of our proposed model for assessing gait in PD with existing models demonstrates a higher level of structural validity in our model than in the existing ones, developed for elderly subjects or patients with PD.

### Inter-trial reliability

We found an excellent reliability for all 16 spatiotemporal variables considered, a good to excellent reliability for the five asymmetric variables and a poor to moderate reliability for many variability variables.

Prior to our study, the only evaluation of inter trial reliability of gait variables measured with the GAITRite^®^ system was performed in healthy subjects [31]. These authors found that inter trial reliability (speed, cadence, stride length, single support and the proportion of the gait cycle spent in double support) was good, with ICCs (3, 1) ranging from 0.85 to 0.97. Even though we assessed a different population (i.e. patients with PD), our inter trial ICC values were comparable to those of [31].

Similarly to Wong et al.’s study [32] in patients with sub-acute stroke, we found a slightly lower ICC value for step width than for cadence, velocity or step length. Variability variables showed low values of ICC, with none reaching an excellent value. Galna et al. [21] assessed reliability of gait variability (SD variables) measured with GAITRite^®^ in patients with PD and found an ICC range between 0.40 and 0.80. According to the latter study, no variability variables (expressed as SD or CV) exceeded an ICC of 0.80. This was confirmed in our study despite some important differences in the procedure of GAITRite^®^ trials acquisition: in Galna et al. [21], patients continuously walked around a 25 m oval circuit within which the walkway located and gait was repeatedly sampled as subjects walked over the walkway. In contrast, we used an intermittent traditional protocol, like almost all studies carried out so far on the GAITRite^®^ walkway [12, 15–18]. Reliability of asymmetry variables had been previously measured only in patients with stroke [33]: in spite of the different disease under study, our findings are similar to those of patients with stroke. Overall, this underlines the reliability of variability variables even in different diseases, at least when assessed with GAITRite^®^ system.

### Exploratory factor analysis

EFA involved a first phase of criteria extraction through PCA. The number of factors was determined on the basis of different criteria. The methods of Kaiser criterion and/or Cattell Scree Test, commonly used, were integrated with Horn’s parallel analysis and MAP. According to all methods of PCA, we divided our eight gait variables into three different factors, a model structure similar to the models of Verghese et al. [16, 17]. In the latter models, however, only spatiotemporal and variability variables were presented. Therefore, these models did not yield any information besides “gait speed” given that all variables considered in the models of Verghese et al. [16, 17] were related to speed. Moreover, these models did not consider “gait asymmetry”, which would be relevant to assess in PD since it is already altered in the first stages of PD and in other neurological diseases [34, 35]. Thingstad et al. [15], after selecting the variables to be considered in the subsequent factor analysis through correlation matrix and KMO measure, found the same three factors as in our model. Nevertheless, since they considered sixteen gait variables instead of eight as in our model, EFA produced a further factor, named “postural control”. Thingstad et al. [15] in this factor included the variables walk ratio, step length and step width, while other authors [13, 14] included step length asymmetry and step width variability. In contrast, in our analysis all these variables were eliminated in the initial phase of selection: step width SD, since it was one of the variables showing lowest reliability, walk ratio since it was derived from other variables, while the other three variables (step length asymmetry, step width and step length SD) showed a correlation that was either too high or too low. Moreover, the step width variability variable (expressed as SD), showed low to moderate reliability also in the literature (ICC = 0.51 and ICC = 0.65 for intermittent and continuous walking, respectively) (Galna et al. [21]). Not unexpectedly, in Lord’s models [13, 14] “postural control” also presented the lowest percentage of variance when compared with the other factors. A further practical point to consider that makes difficult the application of the “postural control” factor in gait models is that many variables that characterize “postural control” cannot be always evaluated or show low reliability if gait is assessed through inertial sensors rather than pressure-sensitive walkways. Morris et al. [20], for example, tried to reproduce the model of Lord et al. [14] and removed the “postural control” factor since step width and step width SD variables could not be measured using body worn monitors. Nevertheless, since Morris et al. [36] reported that the “postural control” factor had been shown to be sensitive to PD, we consider the lack of this factor in our model as a limit.

Finally, considering the model based on body worn monitors [20], they found factors similar to ours (pace, rhythm, asymmetry and variability), with a larger number of variables. We can, therefore, conclude that different instruments (GAITRite^®^ or body worn monitors) may lead to similar conclusion regarding gait factor structure.

### Confirmatory factor analysis

CFA suggested that our model could be a good solution for the evaluation of gait of patients with PD. The values of all indexes showed that, with the modifications made on the basis of MIs, the final model had an excellent fit. CFI and TLI, in fact, reached values above the cut-off of 0.95. It is also important to note that not only the RMSEA value, but also the upper bound of the CI_90_, were below the value of 0.06, considered to be the cut-off for excellent fit.

Other models did not reach convergence when examined using the SEM [12–18]. Previously, however, these models had never been analyzed and/or confirmed through CFA. Only Lord et al. [13] reproduced the model of Verghese et al. [17], but using only EFA. EFA is normally used as an exploratory first step during the development of a measure, but it must be followed by CFA to confirm the factor structure identified by EFA [37].

One of the reasons for the non-convergence of the other existing models [12–18] could be that each had a considerable portion (about 25–60%) of gait variables that proved to be not reliable in our sample of patients with PD, and they also had some variables that were highly inter-related.

In addition, it is important to note that the number of factors identified in previous models was the same or higher than that found in our model. This could be due to the methods used (Kaiser criterion and scree plot) for determining the number of factors. Though used widespread, these methods have been shown to be less accurate than newer methods (parallel analysis and MAP). In particular, some authors used only the eigenvalue > 1 rule (Kaiser criterion) [12, 15], whilst others used both the Kaiser criterion and scree plot [13], and some did not specify any method at all for determining factors [14, 16–18]. The Kaiser criterion, often in conjunction with the scree plot, sometimes overestimates the number of factors, and this could have negative effects on subsequent analysis [24]. On the contrary, parallel analysis and MAP are considered to be the two most accurate methods for factor retention and they outperform Kaiser’s and Cattell’s criteria [26, 27, 38]. In our study, we tried to analyze the structure of the other models by assessing the number of factors to extract not only with the Kaiser criterion, but also with parallel analysis and MAP methods. It is interesting to note that in all studies our analysis revealed a lower number of factors than that reported by the authors. The non-concordance of these results with our data could be due to the low reliability of the variables included in previous models. Furthermore, in four of the models, parallel analysis and MAP confirmed the results obtained with the Kaiser criterion, while in the other three models these analysis produced fewer factors. Finally, no result was obtained in the MAP method when we considered the gait variables proposed by Hollman et al. [12] since the correlations between variables were too high.

#### Three factors summarize our model

The EFA of our model finally selected three factors, labelled according to previous published models as “pace/rhythm”, “variability” and “asymmetry”, which accounted for about 80% of the variance, with a total of only eight spatiotemporal, variability and asymmetry variables.

#### Pace/rhythm factor

The first factor, named “pace/rhythm”, had three variables: gait speed (which represents the pace of gait), step time, and double support time, which explains the rhythmicity of gait in PD.

In most of the existing models, as in ours, the factor with the largest explained variance is the one that includes gait speed [13–18]. Gait speed is the main variable used to describe gait of patients with PD: indeed, a reduction in gait speed is considered a cardinal feature of gait in these patients [39]. Many authors reported that this variable is also one of the strongest predictors of future falls both in patients with PD [1, 40] and in elderly people [41]. Of note, both in animals and humans, gait parameters change as a function of speed even under “normal” conditions [42]; for this reason, it was challenging to decide which variables to retain in our model. We believe that one of the causes for the non-convergence of previous models lies in their having included a plethora of highly correlated variables. In particular, most models included gait speed, step length and cadence or step time, variables that are derived from or strictly related to each other. Including variables that are sums or products of other variables in the same matrix can cause errors of interpretation of the factor analysis [29]. As a consequence, in our model we decided to maintain only step variables instead of stride variables, and step time instead of cadence, as suggested also by Thingstad et al. [15]. Gatesy et al. [43] showed that in bipedal locomotion, particularly in humans, increase of gait speed is achieved more by an increase of stride length than of stride frequency (generally expressed as cadence, that we removed in favour of step time, since they were strictly correlated). This could be due to the fact that gait speed and step length are presumably controlled by the same cortico-basal ganglia circuit, while cadence (i.e. step time) is controlled by brainstem and spinal cord mechanisms [44]. For these reasons and for the strong correlation of gait speed with step length rather than step time, we decided to retain gait speed and step time, but remove step length.

Finally, the double support time variable is represented in the pace/rhythm factor. This variable is reported to be increased in patients with PD, not only in linear walking [34], but also in curved trajectories [45]. Double support time is a phase of gait cycle considered to be a stabilizing component of gait [46]: not unexpectedly, fallers usually have an increased double support time.

#### Variability factor

The second factor, called “variability”, includes features that are not quantifiable in routine clinical observation. The variability factor refers to unsteadiness and arrhythmic pattern of stepping [47]. The increased gait variability and impaired rhythmicity in PD worsen as the PD progresses [45, 48, 49] and may reflect reduced automaticity and damaged locomotor synergies [50, 51]. Increased gait variability is usually associated with an increased fall risk in both elderly individuals [52] and patients with PD [53], suggesting that increased variability may be a very useful element in fall risk assessment. In most gait models presented up to now, the variability variables were dispersed among several factors [13–15]. In many models, the variability was part of the factor in which the variable “walking speed” was present [13–15]. In our study, variability is included as a separate factor from speed: this finding is in accordance with the first studies on gait models in elderly subjects [16–18] and with studies that found gait variability to be influenced independently of gait speed by experimental stressors such as dual task, reduced lighting and walking on a treadmill [54, 55]. In keeping with such independence, it seems that gait speed and gait variability are regulated by different brain functional networks. Exploratory voxel-wise analyses further suggest that gait speed is specifically linked to the functional connectivity of the bilateral middle frontal gyri within the frontoparietal control network [44]. On the other hand, gait variability seems primarily linked to the right superior parietal sulcus within the dorsal attention network [56]. In keeping with different brain networks regulating gait speed and variability, it has been shown that, among gait variability parameters, swing time variability is independent from gait speed both in patients with PD and in healthy subjects [55]. Indeed, swing time variability is determined predominantly by balance-control mechanisms [45, 57]. On the other hand, it seems that other gait variability measures such as step length CV, known to be increased in PD as a function of the Hoehn and Yahr stage [58], are predominantly determined by the gait-patterning mechanism. Moreover, other variability parameters such as stride time variability reflect automatic rhythmic stepping mechanisms and are more sensitive to different rhythmic rates, and hence walking speeds.

#### Asymmetry factor

The third factor of our model, termed “asymmetry”, is composed of two temporal asymmetry variables: step and swing time. Asymmetry of gait has been reported in patients with neurological disorders such as cerebrovascular disease [35, 59] and PD [34, 60–62], as well as in amputees [63]. Specifically for PD, alterations in gait symmetry were observed both in patients with recent diagnosis [34] and in those with mild PD [61] as well as moderate PD [60]. The mechanisms underlying the left–right coordination of walking in PD are poorly understood. In a recent study, Fling et al. [62] suggested that a reduced transcallosal sensorimotor structural connectivity may be a significant mechanism underlying bilateral gait asymmetries in patients with PD, particularly in reference to spatial rather than temporal coordination.

Swing time asymmetry seems to be altered from the early stages of PD and increases more in patients with freezing of gait episodes than in those without [60]. Unexpectedly, this gait variable did not show any association with asymmetry of clinical symptoms, such as tremor and rigidity [60, 61]. Interestingly, in the case of step time asymmetry, no association was found with disease severity or other spatiotemporal variables of gait [64].

### Limitations

Our study has some limitations. The factor analysis was conducted only in a sample of patients with PD, without first analysing the model in a sample of elderly subjects. Hence, further studies are required to verify models of gait specific for elderly individuals. Moreover, even if the model was created to evaluate the general population of patients with PD, we cannot draw conclusions regarding the relationship between cognitive performance and gait since relevant tests were administered only in about 30% of patients.

Our new model is composed by only three factors, without the “postural control” factor: since Morris et al. [36] reported that this factor had been shown to be sensitive to PD progression, we could consider its lack as a limit.

In the future, it might be interesting also to validate our model with accelerometers that offer a series of advantages such as lower cost and applicability in free-living situations. However, right now we believe it is important to have a model usable with electronic walkways since they are still frequently used in scientific studies and clinical practice [7, 65–68].

## Conclusions

In summary, our study showed that a small number of variables can describe a large variance of the gait pattern of patients with PD. The model we identified demonstrated to be more appropriate than existing models, whether developed for elderly or patients with PD. Our model may be useful for future research since clinicians will be able to assess gait of patients with PD and to evaluate improvement following rehabilitation limiting the analysis to the eight variables identified.

Finally, from a methodological point of view, our study also suggests the importance of creating models in which there are no redundant variables and in which methods for determining the number of factors to be extracted are accurately selected.

## Methods

### Participants

Data were collected from 250 patients with idiopathic PD with median Hoehn–Yahr (H&Y) stage 2.5 evaluated at the Scientific Institute of Veruno (Novara, Italy) of the Istituti Clinici Scientifici Maugeri (IRCCS), between February 2014 and April 2017. PD was defined according to the UK Parkinson’s disease Society Brain Bank Criteria [69]. Inclusion criteria were: (a) ability to understand the required motor tasks; (b) ability to walk independently, with or without an assistive device; (c) absence of recent bone fractures. Exclusion criteria were: (a) musculoskeletal injury limiting the ability to walk; (b) any other serious cardio-respiratory problem. The Ethics Committee of the Institute approved the study (approval number # 905 CEC), and informed consent was obtained from all patients. Table 3 summarizes patient’s characteristics: gender, age, height, body mass index (BMI), disease duration, levodopa equivalent daily dose (LEDD) and H&Y stage.

**Table 3:** Clinical details of patients

	Patients (*n* = 250)
Sex	*M* = 134; *F* = 116
Age (years)	69.8 ± 8.7
Height (cm)	164.7 ± 8.7
BMI (kg/m^2^)	26.2 ± 4.4
Disease duration (years)	7.7 ± 5.3
LEDD	660.5 [434.8, 1071.8]
H&Y stage	2.5 [2.0, 3.0]
H&Y 1 (*n*)	5
H&Y 1.5 (*n*)	24
H&Y 2 (*n*)	52
H&Y 2.5 (*n*)	91
H&Y 3 (*n*)	73
H&Y 4 (*n*)	5

Values are expressed as mean ± standard deviation or median and interquartile range [25%, 75%]

### Procedure

Spatiotemporal variables of gait were acquired using a GAITRite^®^ electronic walkway (GAITRite^®^, CIR Systems, Sparta, NJ, USA). Subjects were asked to walk on the walkway barefoot at a comfortable speed; participants started to walk 2 m before and stopped 2 m after the end of the walkway in order to avoid speeding up and slowing down on the walkway. Subjects repeated the walk four times [35], but the first trial was not considered for further analysis. That because the main gait variables of first trial were significantly different from the other three, so it was considered as a practical trial. Based on the recorded footfalls, the walkway calculated several different gait variables. We considered all variables proposed in previous models [12–18] as summarized in Fig. 2. In addition, we averaged all single spatiotemporal and variability variables, the latter ones expressed as SD, from the right and left foot. Finally, for all significant variables we calculated the following measures already considered in previous studies: (a) variability variables expressed as coefficient of variance (CV = SD/mean*100); (b) asymmetry variables calculated as 100*|ln (left/right)|; (c) walk ratio as the ratio step length/cadence.

### Statistical analysis

Statistical analysis was divided into four phases (see Fig. 2 for details): (1) inter trial reliability; (2) correlation matrix; (3) EFA, necessary for creating a new model of gait based on our sample of PD patients; (4) CFA conducted on our new model and on the seven existing models. All analyses were performed using STATA R13.0 statistical software package (StataCorp, College Station, TX, USA).

#### 1. Inter trial reliability

The 2nd, 3rd and 4th walking trial were used to calculate the reliability of the 37 variables collected. For each walking trial, an average value of each single variable was computed from about 7–8 steps. This value was used for the reliability calculation. Reliability was estimated by means of the ICC from three walking trials performed by the 250 subjects. In particular, we used an ICC 3,k type (Model 3, Form k) [70] because the rater was fixed and the data used to calculate the ICC were the mean data of the steps in each walking trial. An ICC < 0.40 was considered as indicating poor reliability, 0.40 ≤ 0.59 moderate reliability, 0.60 ≤ 0.74 good reliability, and ≥ 0.75 excellent reliability [71].

#### 2. Correlation matrix

A correlation matrix was created to display the relationship between variables that showed an excellent reliability. Unlike the inter trial reliability calculation in which the “average values” of three walking trials were used, in the case of the correlation matrix build, only one average value was chosen among the three available values for each variable. The correlation between two variables was then calculated by pairs of 250 average values, one pair for each subject. For EFA (see below), only the variables with correlation coefficients ≥ 0.30 but < 0.90 were considered [72]. Finally, the KMO of sampling adequacy was run: KMO values for each single variable and overall KMO should be > 0.50 and > 0.70, respectively, to be considered acceptable for analysis [29].

#### 3. Exploratory factor analysis

EFA was conducted with the variables selected according to the correlation matrix. EFA is a technique used to determine if numerous measures can be explained by a small number of factors [29]. The extraction of factors was made using PCA (for the sake of clarity, we used the term factor for both PCA and EFA, even if “component” is more appropriate for PCA results) [29]. As suggested by Williams et al. [23], different criteria are necessary to identify the number of factors: (a) Kaiser’s criteria, which considers only factors with an eigenvalue > 1 [73]; (b) Cattell’s Scree Test, a method that involves visual examination of a plot of eigenvalues for each factor: it identifies the breakpoint at which the scree begins; only factors that do not belong to the scree are retained [74]; (c) cumulative percent of variance extracted; (d) Horn’s parallel analysis, in which a random data set is generated and superimposed on the scree plot of real data: only those factors whose eigenvalue is greater than that from random data are retained [75]; (e) MAP rule [76], a method based on the analysis of partial correlation matrices.

Once the number of factors was determined through PCA, EFA proceeded with factor rotation. Factor rotation is commonly conducted in order to assess the distribution of variables into factors, obtaining a solution that is easier to interpret than the initial factor extraction. Since factors were expected to be correlated, a Promax oblique rotation method was used. Promax rotation produces correlation coefficients of items: items with factor loadings higher than 0.32 were considered relevant for that factor [72].

#### 4. Confirmatory factor analysis

Our sample size satisfied by an ample margin the two general criteria for CFA [77]: a minimum of 200 cases and a subject-to-item ratio of 20:1. For CFA, only one average value was chosen from the two that were not used in the build of the correlation matrix.

The structural validity of both our new model of gait and of the existing ones [12–18] was examined with CFA, using SEM. CFA is a model-testing technique in which the hypothetical structure of a measure is tested [78]. In SEM analysis, conducted with the maximum likelihood method, *χ*^2^ test was used to identify whether the model fitted the data well. In addition, the models’ goodness of fit was assessed using the following indices: Comparative Fit Index (CFI) [79], Tucker–Lewis Index (TLI), Root Mean Square Error of Approximation (RMSEA) [80] with 90% confidence interval (CI_90_) and the Standardized Root Mean square Residual (SRMR) [79]. A CFI and TLI > 0.95, RMSEA < 0.06 and SRMR < 0.08 indicate a good fit [81].

We allowed unique variances of different items to correlate in the case models demonstrated an acceptable fit in some indices but not in others. These secondary modifications do not affect conclusions about adequacy of a factor structure, but they can improve model fit by increasing the proportion of variance explained [82]. The selection of which correlations to perform between unique variances were based on the MIs [83]. MIs considered were suggested by SEM group options of STATA 13.0 [84].

On the contrary, when the existing models did not reach convergence at SEM analysis, we investigated their structure. Since in previous studies, the method for determining the number of factors of these existing models was only the Kaiser criterion or was not specified, we assessed the number of factors for each previous model with two more recent methods of extraction: parallel analysis and MAP.

Finally, the goodness of fit of the seven models was compared by computing the *χ*^2^ difference tests of each model pair, calculated as a $\chi^{2} = \, \chi^{2}_{2} - \chi^{2}_{1}$ with d*f* = d*f*_2_ − d*f*_1_.

## Data Availability

The datasets used and/or analysed during the current study are available from the corresponding author on reasonable request.

## Abbreviations

BMI: body mass index
CFA: confirmatory factor analysis
CFI: Comparative Fit Index
CI_90_: 90% confidence interval
CV: coefficient of variation
EFA: exploratory factor analysis
GC: gait cycle
H&Y: Hoehn and Yahr
ICC: intra-class correlation coefficients
KMO: Kaiser–Meyer–Olkin measure
LEDD: levodopa equivalent daily dose
MAP: minimum average partial
MIs: Modification Indices
PCA: principal component analysis
PD: Parkinson’s disease
RMSEA: Root Mean Square Error of Approximation
SD: standard deviation
SEM: structural equation modeling
SRMR: Standardized Root Mean square Residual
TLI: Tucker–Lewis Index
