# Correction to: Proposal of a new conceptual gait model for patients with Parkinson’s disease based on factor analysis

**DOI:** 10.1186/s12938-019-0698-2

**Published:** 2019-07-23

**Authors:** Ilaria Arcolin, Stefano Corna, Marica Giardini, Andrea Giordano, Antonio Nardone, Marco Godi

**Affiliations:** 1 Istituti Clinici Scientifci Maugeri Spa SB (IRCCS), Pavia, Italy; 2 Department of Clinical-Surgical, Diagnostic and Pediatric Sciences, University of Pavia, Pavia, Italy

## Correction to: BioMed Eng OnLine (2019) 18:70 10.1186/s12938-019-0689-3

It was highlighted that the original article [1] had an omission in the Abstract. This Correction article shows the omitted sentence: “This article [1] was awarded the SIAMOC Best Methodological Paper 2018”.

## References

[1] Arcolin I, Corna S, Giardini M, Giordano A, Nardone A, Godi M (2019). Proposal of a new conceptual gait model for patients with Parkinson’s disease based on factor analysis. BioMed Eng OnLine.

